# Neonatal pain

**DOI:** 10.1111/pan.12293

**Published:** 2013-11-13

**Authors:** Suellen M Walker

**Affiliations:** Portex Unit: Pain Research, Department of Anaesthesia and Pain Medicine, UCL Institute of Child Health, Great Ormond Street Hospital for Children NHS Foundation TrustLondon, UK

**Keywords:** pain, neonate, neurodevelopment, NICU, opioids, regional analgesia

## Abstract

Effective management of procedural and postoperative pain in neonates is required to minimize acute physiological and behavioral distress and may also improve acute and long-term outcomes. Painful stimuli activate nociceptive pathways, from the periphery to the cortex, in neonates and behavioral responses form the basis for validated pain assessment tools. However, there is an increasing awareness of the need to not only reduce acute behavioral responses to pain in neonates, but also to protect the developing nervous system from persistent sensitization of pain pathways and potential damaging effects of altered neural activity on central nervous system development. Analgesic requirements are influenced by age-related changes in both pharmacokinetic and pharmacodynamic response, and increasing data are available to guide safe and effective dosing with opioids and paracetamol. Regional analgesic techniques provide effective perioperative analgesia, but higher complication rates in neonates emphasize the importance of monitoring and choice of the most appropriate drug and dose. There have been significant improvements in the understanding and management of neonatal pain, but additional research evidence will further reduce the need to extrapolate data from older age groups. Translation into improved clinical care will continue to depend on an integrated approach to implementation that encompasses assessment and titration against individual response, education and training, and audit and feedback.

## Introduction

Effective and safe management of procedural and postoperative pain is important for children of all ages for humanitarian reasons and to minimize acute physiological and behavioral distress. In addition, reducing pain can improve both acute and long-term outcomes and evidence to guide pediatric clinical practice is increasing (1,2). However, neonates and infants are at increased risk of experiencing moderate to severe pain during hospital care (3,4). Further, specific evidence is required to guide neonatal practice and reduce the need to extrapolate data from older age groups, but implementation of current best practice is also an ongoing challenge. Guidelines and local practice protocols are increasingly available, and although variability in uptake continues to be reported (5), improvements have also been noted; for example, with increased use of opioid analgesia for both procedural and postoperative pain in NICU (6,7) and protocols for safe administration in the ward setting (8).

## Pain mechanisms in the neonatal period

Responses to painful stimuli can be demonstrated in nociceptive pathways from the periphery to the cortex in neonates, although the degree and nature of response change with age. Peripheral pain receptors (nociceptors) respond to mechanical, thermal and chemical stimuli following birth, and peripheral sensitization or primary hyperalgesia (reduced threshold and enhanced response to previously painful stimuli) develops within areas of tissue injury (9).

The spinal cord is an important site for the modulation of nociceptive input but is characterized in early development by a relative excess of excitation and delayed development of local and descending inhibition (10–12). In addition, there are anatomical changes in the distribution of incoming sensory fibers in early development, as A-beta myelinated fibers (that respond to light touch and are restricted to laminae III-IV of the adult dorsal horn) extend into superficial laminae I-II and overlap with A-delta thin myelinated and unmyelinated C-fibers that respond to noxious stimuli (12). As a result, neonatal spinal reflex responses are more generalized, and the threshold is lower (i.e., a reflex response is evoked by a less intense stimulus). Stimulus–response relationships are still evident in human neonates, as noxious heel lance produces a greater reflex withdrawal response than touch (13). Neonatal tissue injury, such as repeated heel lance or inflammation, reduces threshold (i.e., increases sensitivity), but these effects may be minimized by analgesia (14,15).

Pain signals reach the somatosensory cortex in preterm and term neonates. Near-infrared spectroscopy (NIRS) (16,17) and electroencephalogram recordings (18) demonstrate alterations in cortical activity following heel lance for blood sampling. Postnatal age, sleep state, opioid analgesia, and previous experience can also influence the pattern, degree, and latency of response (19–21).

### Acute effects and assessment of neonatal pain

Pain produces a range of physiological and behavioral responses in neonates that can be utilized in clinical assessment tools to quantify pain severity and evaluate analgesic efficacy. A range of validated tools are available for use in different practice settings (1,2,22,23), with some examples described in Table 1 (24–27). Additional measures, such as changes in stress hormones and measures of cortical activity, have been utilized in research settings (9). The adverse impact of inadequate analgesia/anesthesia on acute morbidity following neonatal surgery has long been recognized (28,29).

**Table 1:** Examples of neonatal pain assessment tools

Tool	Parameters	Score	Utility
Premature infant pain profile (PIPP) (24)	Gestational age, behavioral state, heart rate, oxygen saturation, brow bulge, eye squeeze, nasolabial furrow	Total: 0–21 each parameter scored 0–3; ≤6 minimal pain; >12 moderate to severe pain	Procedural and postoperative pain
FLACC (25)	Face, legs, activity, cry, consolability	Total: 0–10 each parameter scored 0–2; >4 moderate pain; >7 severe pain	Procedural and postoperative pain
COMFORT scale (behavioral and physiological parameters) (26)	Alertness, calmness, respiratory distress, movement, muscle tone, facial tension, blood pressure, heart rate	Total: 8–40 each parameter scored 1–5; 17–26 adequate sedation; ≥27 inadequate sedation/analgesia	Pain and sedation in NICU
COMFORT behavior scale (27)	Alertness, calmness, respiratory response (ventilated neonate) or crying (not ventilated), movement, muscle tone, facial expression	Total: 8–30 each parameter scored 1–5; >17 moderate pain requiring intervention	Postoperative pain in NICU

### Long-term effects of neonatal pain

Changing levels of neural activity can alter the normal development of the central nervous system (CNS). As a result, there is increasing awareness of the need to not only reduce acute behavioral responses to neonatal pain, but also to protect from persistent sensitization of pain pathways and potential damaging effects of excess activity on brain development (9,29,30). For example, sucrose effectively reduces the acute behavioral response to painful procedures (31) but does not reduce spinal reflex response or cortical activity (13) or prevent hyperalgesia (32) and therefore may not prevent adverse effects of repeated procedures. Comparative studies with other analgesics are required.

Neonatal surgery has been associated with alterations in future pain response. Following neonatal circumcision without analgesia, the behavioral response to immunization many months later is enhanced (33). Increased perioperative analgesic requirements were noted in infants who had also required surgery as neonates (34). More persistent changes in sensory processing were found in children 8–12 years following neonatal intensive care (35,36), and the degree of change was more marked in those who also required surgery during the neonatal period (37).

Studies in postnatal rodents allow evaluation of the effects of pain and injury at different stages of mammalian development. Age-dependent changes in response to neonatal injury have been demonstrated, with long-term alterations in sensory function that are not seen when the same injury is performed at older ages. Underlying mechanisms and modification by analgesia can also be assessed (9). Altering sensory input into the spinal cord during the neonatal period impairs normal development of both excitatory and inhibitory synaptic function (10,11). Plantar hindpaw incision, an established model of postoperative pain demonstrates differences in the acute and long-term impact of neonatal surgical injury (38–40). Prior neonatal incision effects both excitatory and inhibitory synaptic function (41–43) and increased microglial reactivity in the spinal cord (44) contributes to an enhanced degree and duration of hyperalgesia following subsequent injury. Peripheral nerve block modulates these effects (40,45), and ongoing studies will allow the evaluation of other analgesic interventions.

Adverse neurodevelopmental outcomes following neonatal intensive care are well documented (see Marlow, this edition). Increased exposure to procedural pain has been associated with poorer cognitive and motor scores (46), impairments of growth (47), reduced white matter and subcortical gray matter maturation (48), and altered corticospinal tract structure (49). In addition, poorer neurodevelopmental outcomes have been reported in surgical vs nonsurgical groups following preterm birth (50) and with surgical vs medical management of patent ductus arteriosus (PDA) and necrotizing enterocolitis (51–53). Patients with significant neonatal brain injury are often excluded, and statistical methods are used to correct for potential confounding factors. Specific contributions of pain, analgesia, and anesthesia can be difficult to determine, and respiratory disease (54) and hypotension (48,55) make independent contributions.

## Opioid analgesia

### Pharmacokinetics and mechanisms

Intravenous opioid requirements during intensive care management (56) and the postoperative period (8,57) are lower in neonates than in infants and children. Pharmacokinetic parameters are influenced by age and clinical state, with decreased clearance in neonates, and additional variability following cardiac surgery and with changes in organ function and blood flow (e.g., reduced hepatic blood flow and morphine clearance with positive pressure ventilation) (58,59). A recent model based on bodyweight was able to predict clearance across all age ranges, including neonates (60). Laboratory studies also document age-and dose-dependent changes following systemic (61), epidural (62,63), or intrathecal (64) administration and allow evaluation of associated pharmacodynamic changes. Altered opioid receptor distribution and density in the dorsal root ganglion and spinal cord contribute to increased sensitivity (61,65,66) and are not solely related to changes in blood–brain barrier permeability for morphine (63) as an increased effect for the same CNS tissue concentration is present at younger ages (67).

### Analgesic efficacy

Intravenous opioid infusions have an established role for perioperative analgesia in neonates (1). Protocols vary and include continuous infusions, intermittent bolus doses, or nurse-controlled analgesia (NCA) (8,57,68). NCA is delivered via the same type of pump as patient-controlled analgesia, with a prescribed bolus and dose interval, but addition of background infusions in opioid-naïve neonates may increase the risk of respiratory depression (8,68)). Examples of local NCA protocols can be found at http://www.gosh.nhs.uk/health-professionals/clinical-specialties/pain-control-service-information-for-health-professionals/download-documentation/. Protocols need to be sufficiently flexible to allow for interindividual variability and titration against individual response, with regular assessment of pain score, efficacy and side-effects. Morphine and fentanyl are most often used. There is limited specific data to guide remifentanil dosing in neonates (69,70), but use in NICU for analgesia and sedation (71), perioperative analgesia (72), and intubation (73) has been reported, and the short duration of action may be advantageous for procedural pain management in NICU (74,75).

### Side effects

Fear of side effects, particularly respiratory depression, has contributed to inadequate use of opioids in neonates. Large audits have demonstrated higher rates of opioid-induced respiratory depression in neonates than in older children (2.5% vs 0.27%), but long-term sequelae are rare with appropriate monitoring and management (8,68). Overall, doses did not differ between neonates with or without respiratory depression, but risk was increased by preterm birth and intercurrent comorbid conditions (8,68).

Opioid withdrawal in neonates is a significant problem following maternal opioid use during pregnancy (neonatal abstinence syndrome) and is also associated with significant neurodevelopmental impairment (76,77). However, iatrogenic opioid tolerance and withdrawal symptoms also occur in neonates, particularly with prolonged use, continuous rather than intermittent administration, and shorter acting agents such as fentanyl (70,78,79). Assessment tools and management protocols are available (78,80).

### Long-term effects of neonatal opioids

Evaluating the long-term impact of pain and analgesia in clinical cohorts is dependent on correction for clinical confounders and will also be influenced by the sensitivity of the outcome measure and age at follow-up. Following NICU, greater overall exposure to intravenous morphine was associated with poorer motor development at 8 months, but not at 18 months (46). Associations between routine use of morphine for sedation during mechanical ventilation and poor neurodevelopmental outcome (81) have not been confirmed in all analyses (55,82,83), and there is often limited data on indication (e.g., sedation, procedural, or postoperative pain), and variability in dose and duration of therapy (84). Initial follow-up of mechanically ventilated neonates at 5 years of age suggested an impairment on one component (visual analysis) of the IQ test (85), but subsequent evaluation of neuropsychological outcomes in the same population at 8–9 years reported no impairment related to neonatal morphine use (86).

Associations between exposure to general anesthesia in early life, increased levels of neuronal apoptosis (programmed cell death), and impaired neurodevelopmental outcomes have been demonstrated in a number of mammalian species (87–89). Laboratory models have also evaluated the impact of neonatal opioid exposure on neuronal apoptosis, but it is important to differentiate dose schedules associated with the development of dependence and tolerance (which may be relevant to prolonged NICU care) from perioperative analgesic dosing. Subcutaneous morphine 0.3–1.0 mg·kg^−1^ produces analgesia in neonatal rats (61). When tolerance is induced by subcutaneous morphine 10 mg·kg^−1^ bd from postnatal day P1 to P7 (90), neuronal apoptosis is increased in the cortex and amygdala, but not in regions important for memory (hippocampus) or nociceptive processing (periaqueductal gray, PAG) (91). In adult rats, repeated *intrathecal* morphine (0.03 mg·kg^−1^ bd for 7 days) produces tolerance and increases apoptosis in the spinal cord (92). However, in neonatal rodents, single doses of morphine up to 3 mg·kg^−1^ (300 times the analgesic dose of 0.01 mg·kg^−1^ at this age) did not increase apoptosis or produce any long-term impairment of spinal function, measured by sensory reflex thresholds and gait analysis (64). Whereas general anesthesia for 4 h with isoflurane, nitrous oxide, and midazolam increased cortical apoptosis in the neonatal piglet (93) and guinea pig (94), no significant increase was seen in sham control groups given fentanyl (30 mcg·kg^−1^ bolus and 4 h infusion 15 mcg·kg^−1^·h^−1^). Daily subcutaneous morphine 0.5 mg·kg^−1^ from P1 to P3 or P1 to P5 did not alter levels of apoptosis in the brain. When combined with a pain stimulus (daily paw injection of formalin), this analgesic dose of morphine reduced injury-related apoptosis in the P1–P3 group, but not in the more prolonged pain group (P1–P5 formalin plus morphine) (95). Further studies are required to evaluate relationships between opioids and injury-induced apoptosis and investigate other mechanisms influencing neurodevelopmental outcomes.

## Paracetamol

### Pharmacokinetics and mechanisms

The pharmacokinetic profile of paracetamol in neonates has been evaluated following rectal (96), intravenous (97) and repeat IV doing over 4 days (98), and issues of neonatal dosing discussed (99). Clearance is related predominantly to weight (57% of variance), and age between postmenstrual age 28–44 weeks has minimal effect (2.2% variance) (97). A 20 mg·kg^−1^ loading and 10 mg·kg^−1^ IV dose every 6 h was predicted to achieve a serum concentration of 11 mg·L^−1^ in neonates (32–44 weeks PMA), although it was noted that safety data for this dose and drug are limited in neonates (97).

Different mechanisms contribute to the analgesic effect of paracetamol (see recent reviews (100,101)), including:

1. Prostaglandin-mediated effects, as despite the limited peripheral anti-inflammatory action compared with NSAIDs, central effects may relate to interaction with different cyclo-oxygenase sites (102).
2. The metabolite N-arachydonylphenolamine (AM404) is a ligand for the cannabinoid CB1 receptor and an uptake inhibitor of anandamide (an endogenous cannabinoid) (103).
3. Interaction with serotonergic mechanisms enhances inhibitory pathways descending from the brainstem to the spinal cord (104).
4. Effects on the spinal neurotransmitter nitric oxide.

Dose-dependent analgesic efficacy and specific spinal cord-mediated effects have been demonstrated in adult animal models (105,106), but further evaluation of dose response and mechanisms during postnatal development is warranted.

### Analgesic efficacy

Analgesic efficacy of paracetamol is influenced by dose, route of administration, and type of pain stimulus. Oral paracetamol 20 mg·kg^−1^ in neonates did not reduce the behavioral response to heel prick (107). Intravenous paracetamol (20 mg·kg^−1^ loading, 5–10 mg·kg^−1^ 6-h, and 20–40 mg·kg^−1^ per 24 h maximum) was effective for moderate pain in neonates in NICU, producing a significant trend to lower pain scores at 30 min, with a slight decrease in effect by 5–6 h (108). In the perioperative setting, multimodal analgesia with addition of paracetamol to opioid regimes can reduce opioid requirements and/or improve analgesia (109). Rectal paracetamol (30–40 mg·kg^−1^ loading and 20 mg·kg^−1^ 6–8 h) did not reduce NCA opioid requirements in neonates and infants following major surgery, although marked variability in plasma concentration following rectal dosing was noted (110). A recent study from the same group noted a significant reduction in opioid requirements in neonates and infants following major surgery with intravenous paracetamol (30 mg·kg^−1^ per day in four doses) (111). Current recommended doses for intravenous paracetamol in term neonates are 7.5 mg·kg^−1^ 6-h with a maximum daily dose of 30 mg·kg^−1^ (1) (http://www.rcoa.ac.uk/system/files/intravenousparacetamol.pdf). Ongoing studies and monitoring are required to further evaluate analgesic dose response and safety in neonates.

Recent case series have reported an association between use of IV paracetamol 60 mg·kg^−1^·d^−1^ for 3–6 days and PDA closure in preterm neonates (112–114). Although significant morbidity may be associated with the alternative treatment (indomethacin/ibuprofen or surgical closure), caution with these high doses of paracetamol is also warranted, and there is no confirmed mechanism for this effect of paracetamol that allows prediction of the appropriate dose (113).

### Side effects

In preterm and term neonates undergoing procedures in NICU, intravenous paracetamol 10 or 20 mg·kg^−1^ produced statistically significant but modest reductions in heart rate (average 7 b·min^−1^ at 30–120 min) and blood pressure (3 mmHg at 60 min). Changes were more marked in neonates with preexisting hypotension, suggesting impaired hemodynamics may be a relative contraindication to IV paracetamol (115).

Paracetamol overdose and hepatotoxicity has been reported in neonates (99,116,117) and infants (118). Awareness of the risk of accidental administration of milliliter rather than milligram resulting in a 10× overdose, and use of smaller intravenous paracetamol vials for neonates, has been highlighted by The UK Medicines and Healthcare Products Regulatory Agency (MHRA; http://www.mhra.gov.uk/Safetyinformation/DrugSafetyUpdate/CON088171).

Mechanisms of paracetamol toxicity are discussed in recent reviews (99,113). Briefly, oxidative metabolism of paracetamol (normally 5–10% vs. 50–60% glucuronidation and 25–30% sulfation) results in formation of the intermediate N-acetyl-p-benzoquinone imine (NAPQI). Although usually conjugated to glutathione and excreted in the bile, paracetamol overdose or glutathione lack results in accumulation of NAPQI and toxicity due to apoptosis and necrosis of hepatocytes. Although a reduced rate of oxidation and increased ability to replete glutathione may be protective for neonates (119), there is little data on relationships between paracetamol dose and NAPQI levels, or capacity for NAPQI detoxification in neonates, and immaturity of hepatic transporters or poor nutritional status may increase susceptibility (99,113).

### Long-term effects

An epidemiologic link has been reported between paracetamol use in early life and increased risk of asthma in childhood (120,121). Others have reported that an increased number of respiratory infections, rather than paracetamol *per se*, is the important contributing factor (122,123). Differentiating association and causation is also difficult as the proportion of infants and children who receive paracetamol is high: 51% by 12 weeks of age and 97% by 2 years in a cohort with a family history of allergy (123); and over 1% before 8 weeks age and almost 95% by 4.5 years in a UK cohort (124).

## Regional analgesia

### Techniques and efficacy

A range of regional analgesic techniques can be effectively used in neonates (see recent reviews plus special edition of Pediatric Anesthesia January 2012) (125–127). Although analgesic efficacy has been demonstrated for many, there has been limited direct comparison of techniques or evaluation of relative benefits and risks in controlled trials in neonates (1). Dorsal penile nerve block was more effective than topical local anesthetic for circumcision performed in awake neonates (128). Ilio-inguinal and rectus sheath blocks are the commonest intraoperative regional blocks for neonates (129), transversus abdominus plane (TAP) blocks are feasible (130), and local anesthetic wound infiltration is commonly performed, but additional larger studies are required to confirm benefit (131). Additional benefit may be gained if long-acting local anesthetic preparations are shown to be effective and safe in neonates (132–134).

Spinal/intrathecal, epidural, and caudal routes are utilized for neuraxial anesthesia and/or analgesia in neonates (127,129,135,136). Potential advantages include reducing general anesthetic and opioid requirements, and case series report a reduction in the need for postoperative mechanical ventilation and specific benefit for neonates susceptible to respiratory complications (127,137).

### Complications

Large series from the United Kingdom (138), Europe (129), United States (139), and Canada (140) demonstrate low complication rates following neuraxial analgesia in children, but rates are higher for central vs peripheral blocks (129). In early series, neonates were at greater risk and also had worse outcomes (141,142). Recent series have also reported higher complication rates and more pump programming errors in neonates (129,138,140), thus emphasizing the need for careful monitoring and follow-up.

Prolonged general anesthesia in neonatal rodents increases apoptosis in the spinal cord as well as the brain (143,144). This, plus the lack of systematic data evaluating spinal analgesic toxicity in early development has emphasized the need for preclinical evaluation of spinally administered drugs (127,145,146). No histological injury or increased apoptosis was found following spinal anesthesia with bupivacaine (143) or levobupivacaine (147) in neonatal rodents. Maximum tolerated doses of intrathecal morphine and clonidine (up to 300 times the analgesic dose) did not alter spinal cord histology or function (64,148). By contrast, analgesic doses of intrathecal ketamine increased apoptosis and altered long-term sensory function (149). Although no adverse effects directly related to caudal additives have been reported, there has been limited follow-up in clinical trials. As a result of adverse histological effects in both neonatal and adult animals following neuraxial delivery (150), clinical use of caudal ketamine has reduced (136,145,151).

## Future directions

Significant advances continue to be made in the understanding and management of neonatal pain. Factors that may contribute to further improvements include:

1. Increased high-quality evidence from neonatal trials rather than reliance on extrapolation of doses and techniques from older age groups (1). As ethical and organizational difficulties make recruitment difficult, and samples may be small and heterogeneous, multicentre trials may be required.
2. Availability of pharmacokinetic and pharmacodynamic data from clinical and laboratory studies will continue to inform age-and injury-specific dosing (59,152).
3. Concerns regarding acute side effects have limited use of analgesia in the past, but improvements in monitoring and protocols for safe delivery have significantly improved clinical utility.
4. Additional direct comparison of analgesic techniques in neonates will further delineate the relative safety and efficacy of different drugs and techniques, particularly as the developing nervous system responds differently to pain, anesthesia, and analgesia, and potential adverse impacts on neurodevelopmental outcome may also differ (9).
5. Increased validation and use of neonatal pain assessment tools have improved clinical practice and facilitated titration of analgesia against individual response. However, these tools necessarily rely on observer assessment of behavioral and/or physiological responses as proxy measures of pain and are less specific and sensitive than assessment at older ages (23) (4). A range of neurophysiological and hormonal measures are being evaluated in research studies and may provide useful comparative data in the future.

Importantly, in addition to the above measures, improvements in clinical practice are critically dependent on implementation of current best evidence. An integrated approach is required (153), with targeted education and practice interventions, use of validated assessment tools, local protocols for analgesic administration, and regular audit and feedback, to ensure translation into improved outcomes for neonates requiring anesthesia, surgery, and intensive care.
